# Fairness in Predicting Cancer Mortality Across Racial Subgroups

**DOI:** 10.1001/jamanetworkopen.2024.21290

**Published:** 2024-07-10

**Authors:** Teja Ganta, Arash Kia, Prathamesh Parchure, Min-heng Wang, Melanie Besculides, Madhu Mazumdar, Cardinale B. Smith

**Affiliations:** 1 Division of Hematology and Medical Oncology, Icahn School of Medicine at Mount Sinai, New York, New York; 2 Department of Anesthesiology, Perioperative and Pain Medicine, Icahn School of Medicine at Mount Sinai, New York, New York; 3 Institute for Healthcare Delivery Science, Icahn School of Medicine at Mount Sinai, New York, New York; 4 Tisch Cancer Institute, Icahn School of Medicine at Mount Sinai, New York, New York

## Abstract

**Importance:**

Machine learning has potential to transform cancer care by helping clinicians prioritize patients for serious illness conversations. However, models need to be evaluated for unequal performance across racial groups (ie, racial bias) so that existing racial disparities are not exacerbated.

**Objective:**

To evaluate whether racial bias exists in a predictive machine learning model that identifies 180-day cancer mortality risk among patients with solid malignant tumors.

**Design, Setting, and Participants:**

In this cohort study, a machine learning model to predict cancer mortality for patients aged 21 years or older diagnosed with cancer between January 2016 and December 2021 was developed with a random forest algorithm using retrospective data from the Mount Sinai Health System cancer registry, Social Security Death Index, and electronic health records up to the date when databases were accessed for cohort extraction (February 2022).

**Exposure:**

Race category.

**Main Outcomes and Measures:**

The primary outcomes were model discriminatory performance (area under the receiver operating characteristic curve [AUROC], F1 score) among each race category (Asian, Black, Native American, White, and other or unknown) and fairness metrics (equal opportunity, equalized odds, and disparate impact) among each pairwise comparison of race categories. True-positive rate ratios represented equal opportunity; both true-positive and false-positive rate ratios, equalized odds; and the percentage of predictive positive rate ratios, disparate impact. All metrics were estimated as a proportion or ratio, with variability captured through 95% CIs. The prespecified criterion for the model’s clinical use was a threshold of at least 80% for fairness metrics across different racial groups to ensure the model’s prediction would not be biased against any specific race.

**Results:**

The test validation dataset included 43 274 patients with balanced demographics. Mean (SD) age was 64.09 (14.26) years, with 49.6% older than 65 years. A total of 53.3% were female; 9.5%, Asian; 18.9%, Black; 0.1%, Native American; 52.2%, White; and 19.2%, other or unknown race; 0.1% had missing race data. A total of 88.9% of patients were alive, and 11.1% were dead. The AUROCs, F1 scores, and fairness metrics maintained reasonable concordance among the racial subgroups: the AUROCs ranged from 0.75 (95% CI, 0.72-0.78) for Asian patients and 0.75 (95% CI, 0.73-0.77) for Black patients to 0.77 (95% CI, 0.75-0.79) for patients with other or unknown race; F1 scores, from 0.32 (95% CI, 0.32-0.33) for White patients to 0.40 (95% CI, 0.39-0.42) for Black patients; equal opportunity ratios, from 0.96 (95% CI, 0.95-0.98) for Black patients compared with White patients to 1.02 (95% CI, 1.00-1.04) for Black patients compared with patients with other or unknown race; equalized odds ratios, from 0.87 (95% CI, 0.85-0.92) for Black patients compared with White patients to 1.16 (1.10-1.21) for Black patients compared with patients with other or unknown race; and disparate impact ratios, from 0.86 (95% CI, 0.82-0.89) for Black patients compared with White patients to 1.17 (95% CI, 1.12-1.22) for Black patients compared with patients with other or unknown race.

**Conclusions and Relevance:**

In this cohort study, the lack of significant variation in performance or fairness metrics indicated an absence of racial bias, suggesting that the model fairly identified cancer mortality risk across racial groups. It remains essential to consistently review the model’s application in clinical settings to ensure equitable patient care.

## Key Points

**Question:**

Can a machine learning (ML) model be used to fairly and accurately predict cancer mortality across racial subgroups?

**Findings:**

This cohort study including 43 274 patients with solid malignant tumors calculated fairness metrics to evaluate an ML model for predicting cancer mortality and found no statistical evidence of racial bias in the model’s performance.

**Meaning:**

The findings suggest that assessment for racial bias is feasible and should be a routine part of predictive ML model development and continue through the implementation process.

## Teaser

This cohort study evaluates whether racial bias exists in a machine learning model that identifies cancer mortality risk among patients with solid malignant tumors.

## Introduction

Machine learning (ML) models can transform cancer care by providing oncologists with more accurate and accessible information to augment clinical decisions. There are a variety of use cases for incorporating ML in oncology.^1,2^ Implementation of a mortality predictive model integrated into a decision support system that encourages clinicians to discuss end-of-life care with patients improved rates of these conversations.^3^

A barrier to implementation is the potential role of bias that can exacerbate existing disparities and reflects the impact of structural racism in health care.^4,5,6,7^ For example, patients from minoritized racial and ethnic groups have poor access to health care,^8^ resulting in low sample sizes or missing or incomplete data for training datasets for ML. The use of models based on data lacking detail may further propagate inequities. Such models may demonstrate promising performance in the overall population but fail to meet standards for specific subgroups. While it is possible to create multiple models for each group, small sample sizes for specific groups may not allow for comprehensive implementation. Because race is socially constructed, its inclusion as a variable within prediction tools may lead to unwanted effects, including perpetuation of disparities.^9,10^ Conversely, excluding race may overlook important factors, such as social determinants of health, and continue to bias the algorithm.

When developing an ML model, it is important to evaluate for fairness using a variety of methods. President Biden’s executive order on artificial intelligence (AI) highlighted the importance of developing AI tools that do not contribute to discrimination in health care.^11^ Approaches include examining the data used in development to identify issues in subgroup data quality that may limit model generalizability; measuring performance metrics to ensure there are no between-group discrepancies^8^; and calculating fairness metrics, which provide additional measurements of the extent of bias,^12^ with the most common metrics being equal opportunity, equalized odds, and disparate impact. Equal opportunity asserts that the true-positive rates (TPRs) between groups should be equal. Equalized odds specifies that the TPRs and the false-positive rates (FPRs) between groups should be equal. Disparate impact measures whether the positivity rate (ie, percentage of predictive positive [PPP]) is equal between groups.^12^

Oncologists may incorporate a predictive model in decision-making to identify patients for serious illness conversations (SICs), which occur between clinicians and patients and/or family members to elicit patients’ values, preferences, and goals for medical care.^13^ Patients with cancer who engage in SICs often cite a better quality of life and goal-concordant care.^14^ However, most patients from minoritized racial and ethnic groups who have cancer die without a documented conversation.^15^ Oncology practitioners have identified many barriers to SICs, with patient and familial factors—such as difficulty accepting a poor prognosis and lack of agreement on decision-making—being among the most common.^16^ Uncertainty in estimating prognosis is another contributor; clinicians cannot identify patients at risk of short-term mortality using existing tools and often overestimate prognosis.^17^ Prognostic uncertainty, racial bias, and structural racism may lead clinicians to assume that patients from minoritized racial and ethnic groups do not want to have these conversations.^18^ Machine learning has the potential to help clinicians prognosticate, which can help prioritize patients for SICs. Caution is warranted in building models that do not exacerbate existing bias.

This study describes assessments to identify racial bias (measurable statistical variation in model performance and/or fairness metrics across racial groups) during the development of an ML model that predicts mortality among patients with solid tumors. The model was developed with intention for clinical use to identify patients for SICs, and the racial bias assessments were an important step to ensure safety prior to implementation.

## Methods

### Data Sources and Patient Selection

Patients were included in this cohort study if they had a date of cancer diagnosis recorded in the Mount Sinai Health System (MSHS) cancer registry between January 2016 and December 2021, were 21 years of age or older, and received care at an ambulatory cancer clinic within the MSHS. Data were obtained by matching records from the MSHS cancer registry, Social Security Death Index, and electronic health record and included all available retrospective data up to the date when databases were accessed for cohort extraction (February 2022). Our study followed the Transparent Reporting of a Multivariable Prediction Model for Individual Prognosis or Diagnosis (TRIPOD) reporting guideline.^19^ The MSHS institutional review board approved this study and waived informed consent due to minimal risk based on the protected health information that was accessed.

The MSHS cancer registry is part of the national cancer registry; all data were abstracted by certified tumor registrars to adhere to the North American Association of Central Cancer Registries’ standards.^20^ The Social Security Death Index was obtained from the Social Security Administration’s death information master file and was used to calculate the date of death. Variables included were admission-discharge-transfer events, laboratory results, assessments from nursing flow sheets, cancer stage, and cancer treatment plans (the eTable in Supplement 1 shows the full list). Cancer stage and status and patients’ race and ethnicity were obtained from the cancer registry. Race categories were Asian, Black, Native American, White, and other (not broken down further in the database accessed) or unknown, and ethnicity categories were Hispanic, non-Hispanic, unknown, and missing. Cancer stage was specified as the stage at the time of incident cancer diagnosis. Cancer status was defined as present or not currently detectable (cancer was cured or in complete response from treatment). Race and ethnicity were recorded by certified tumor registrars as part of routine operations. Based on data availability and interval of measurements, different sampling logics were used to extract measurements for each variable (the eTable in Supplement 1 includes the number of measurements sampled). Missing values were imputed by using the median value of the variable over the entire cohort at the time of sampling.

### Model Creation

The target variable was 180-day mortality from the date of prediction. The period for prediction was set as 6 months leading up to the most recent recording of the patient’s vital status (eg, if the patient’s last recording was in December 2021, the prediction period was defined as from June to December 2021). For the retrospective validation cohort, each patient received 1 prediction. To accentuate the differences in data between deceased and living patients, we structured the death case profiles using data from the 30 days preceding death. This strategy was implemented to reduce the cosine similarity in the data representation, thereby enhancing the ML process during training. Structuring the profiles differently for records of alive vs dead individuals was chosen because the objective of the model was to predict the patient’s chance of dying at any time within the next 180 days rather than to predict patient status specifically at day 180.

The dataset was randomly split into the training dataset (70%) and the test dataset (30%). Within the preliminary dataset, it was noted that there was a higher cumulative prevalence of records with a status of alive compared with dead. There were concerns that a training dataset that overrepresented a class in the target variable may lead to model underperformance. To manage this, the training dataset was adjusted using random undersampling; we randomly removed excess records of the alive patients until both classes in the target variable were equally balanced. 10-fold cross-validation was used to train the model by using the random forest algorithm from the open-source Apache Spark project ML library, and recursive feature elimination was used for feature selection.^21,22^ The importance of each feature was calculated using the Gini coefficient. Variable importance is the sum of the Gini coefficients for each measurement of a variable.

### Model Assessment

The primary outcomes for the study were discriminatory performance and fairness metrics among each race category (Asian, Black, Native American, White, and other or unknown) in the test dataset. Overall model discriminatory performance was measured using the F1 score and the area under the receiver operating characteristic curve (AUROC).^23,24^ Fairness metrics were equal opportunity, equalized odds, and disparate impact. Based on consensus among the clinical operational leadership (including C.B.S.) who stewarded the planned implementation of the tool, it was prespecified that a threshold of 80% (ie, a fairness metric ratio between 0.80 and 1.25 [1/0.8]) was evidence of no racial bias. We also considered stricter thresholds of 90% (ratios between 0.90 and 1.11) as a sensitivity analysis.

### Statistical Analysis

Descriptive statistics were used to evaluate patient characteristics in the test cohort. The predicted mortality was compared with the actual mortality. Equal opportunity ratio was calculated as the ratio of TPRs. Equalized odds ratio was both the ratio of TPRs and the ratio of FPRs. Disparate impact ratio was the ratio of the positivity rate. Ratios of TPRs, FPRs, and PPPs and their 95% CIs were computed. The formula used for 95% CIs was exp(ln[ratio of proportions] ± [*Z* × SE]). All statistical calculations were performed using Python, version 3.9.13 (Python Software Foundation).

## Results

The test set consisted of 43 274 patients, of whom 88.9% were alive and 11.1% were dead. A total of 49.6% of patients were older than 65 years (mean [SD] age, 64.09 [14.26] years); 53.3% were female and 46.7%, male. A total of 9.5% were Asian; 18.9%, Black; 0.1%, Native American; 52.2%, White; 19.2%, other or unknown race; and 0.1% had missing race data. A total of 9.6% were Hispanic and 83.6%, non-Hispanic; 6.7% had unknown ethnicity, and 0.1% had missing ethnicity data (Table 1). The Native American cohort was small (n = 45) and was not included in subsequent subgroup analysis. The most common cancers were breast (20.3%), genitourinary (19.9%), or gastrointestinal (19.1%). Among patients who were dead compared with those who were alive, there was a higher proportion of patients who were older than 65 years (63.8% vs 47.8%), were Black (25.2% vs 18.1%), and had gastrointestinal (34.7% vs 17.0%), hematologic (11.6% vs 6.2%), and thoracic (8.2% vs 5.4%) cancers.

**Table 1.** Characteristics in the Test Set

Characteristic	Patients, No. (%)			SMD
	Overall	Alive	Deceased	
Patients	43 274 (100)	38 476 (88.9)	4798 (11.1)	NA
Age, y				
Mean (SD)	64.09 (14.26)	63.44 (14.21)	69.32 (13.57)	NA
<45	4330 (10.0)	4094 (10.6)	236 (4.9)	0.347
45-65	17 476 (40.4)	15 977 (41.5)	1499 (31.2)	
>65	21 468 (49.6)	18 405 (47.8)	3063 (63.8)	
Sex				
Female	23 050 (53.3)	20 872 (54.2)	2178 (45.4)	0.178
Male	20 224 (46.7)	17 604 (45.8)	2620 (54.6)	
Race				
Asian	4112 (9.5)	3739 (9.7)	373 (7.8)	0.188
Black	8180 (18.9)	6971 (18.1)	1209 (25.2)	
Native American	42 (0.1)	33 (0.1)	9 (0.2)	
White	22 580 (52.2)	20 312 (52.8)	2268 (47.3)	
Other or unknown^a^	8301 (19.2)	7365 (19.1)	936 (19.5)	
Missing	59 (0.1)	56 (0.1)	3 (0.1)	
Ethnicity				
Hispanic	4166 (9.6)	3564 (9.3)	602 (12.5)	0.128
Non-Hispanic	36 169 (83.6)	32 219 (83.7)	3950 (82.3)	
Unknown	2878 (6.7)	2635 (6.8)	243 (5.1)	
Missing	61 (0.1)	58 (0.2)	3 (0.1)	
Cancer status				
Evidence of tumor	19 120 (44.2)	15 455 (40.2)	3665 (76.4)	0.838
No evidence of tumor	19 087 (44.1)	18 358 (47.7)	729 (15.2)	
Unknown	2121 (4.9)	1862 (4.8)	259 (5.4)	
Missing	2946 (6.8)	2801 (7.3)	145 (3.0)	
SEER stage				
In situ	1789 (4.1)	1725 (4.5)	64 (1.3)	0.561
Localized	6868 (15.9)	6319 (16.4)	549 (11.4)	
Regional	3115 (7.2)	2604 (6.8)	511 (10.7)	
Distant	3000 (6.9)	2078 (5.4)	922 (19.2)	
Unstaged	1672 (3.9)	1341 (3.5)	331 (6.9)	
Not applicable	616 (1.4)	588 (1.5)	28 (0.6)	
Missing	26 063 (60.2)	23 694 (61.6)	2369 (49.4)	
Cancer types per patient, No.				
1	40 792 (94.3)	36 445 (94.7)	4347 (90.6)	0.026
2	2353 (5.4)	1934 (5.0)	419 (8.7)	
≥3	129 (0.3)	97 (0.3)	32 (0.7)	
Cancer type				
Breast	9338 (20.3)	8988 (22.1)	350 (6.1)	0.543
Genitourinary	9131 (19.9)	8499 (20.9)	632 (12.0)	
Gastrointestinal	8761 (19.1)	6929 (17.0)	1832 (34.7)	
Hematologic	3119 (6.8)	2504 (6.2)	615 (11.6)	
Gynecologic	2823 (6.1)	2503 (6.2)	320 (6.1)	
Thoracic	2647 (5.8)	2212 (5.4)	435 (8.2)	
Head and neck	2538 (5.5)	2276 (5.6)	262 (5.0)	
Endocrine	1976 (4.3)	1927 (4.7)	49 (0.9)	
Neurologic	1964 (4.3)	1765 (4.3)	199 (3.8)	
Skin	1242 (2.7)	1160 (2.9)	82 (1.6)	
Soft tissue	323 (0.7)	267 (0.7)	56 (1.1)	
Bone	96 (0.7)	86 (0.2)	10 (0.2)	
Other	1984 (4.3)	1542 (3.8)	442 (8.4)	

Abbreviations: NA, not applicable; SEER, Surveillance, Epidemiology, and End Results program; SMD, standardized mean difference.

^a^ Other race was not broken down further in the database accessed.

**Figure 1.**
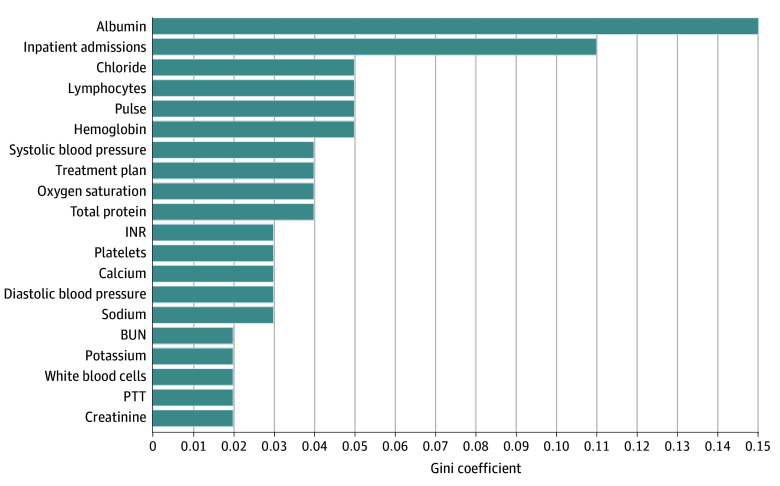
Top 20 Most Important Features in the Predictive Model Using the Gini Coefficient BUN indicates blood urea nitrogen; INR, international normalized ratio; PTT, prothrombin time.

**Figure 2.**
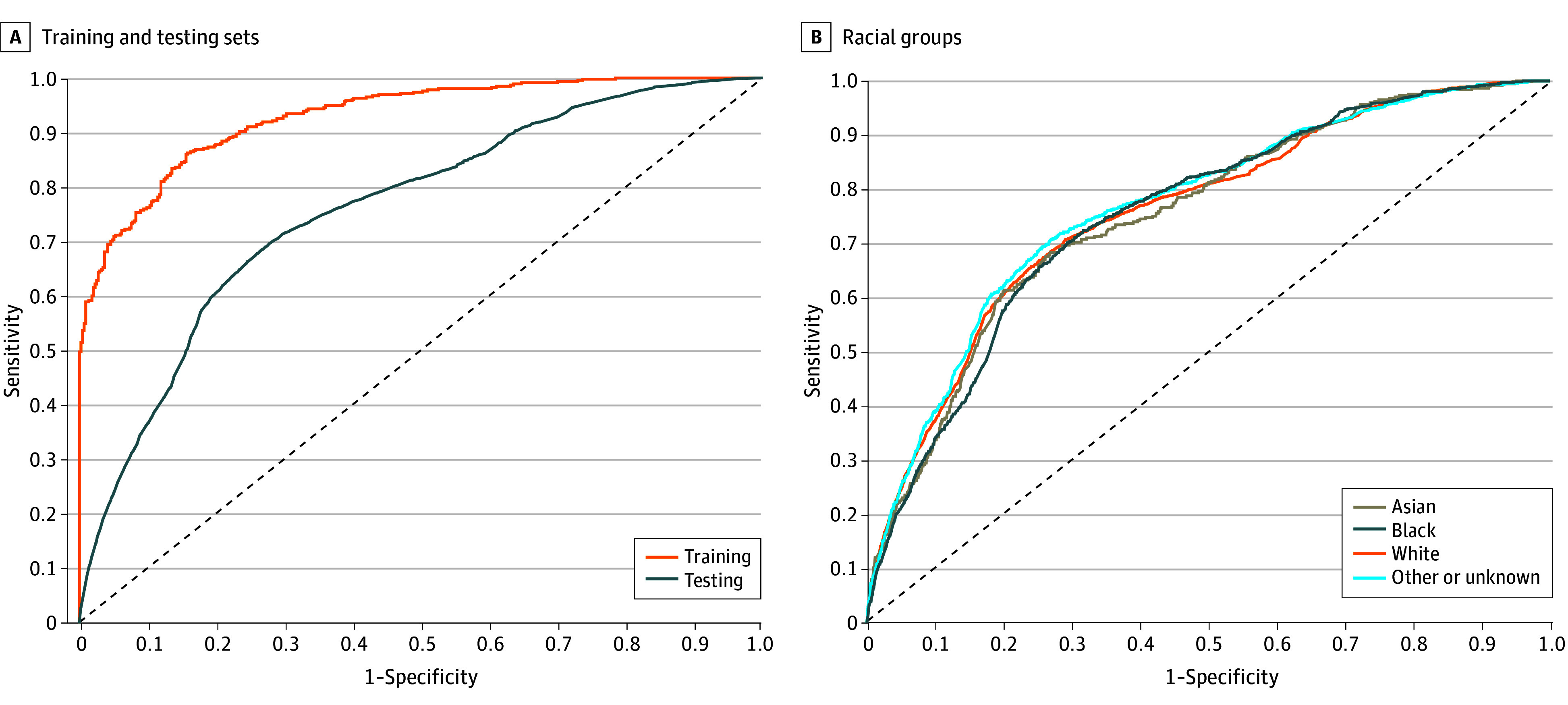
Receiver Operating Characteristic (ROC) Curves A, The area under the curve (AUC) for testing was 0.93 and for training was 0.76. B, The AUC for Asian individuals was 0.75; for Black individuals, 0.75; for White individuals, 0.76; and for individuals of other or unknown race, 0.77.

There were 35 variables in the model. The most important as ranked by Gini coefficient were albumin, number of inpatient admissions, chloride, lymphocyte percentage, and heart rate (Figure 1). For cancer-specific variables, treatment plan ranked as the eighth most important. Cancer stage was not in the top 20 most important variables. Overall, the model performed with a sensitivity of 0.71 (95% CI, 0.69-0.73), specificity of 0.71 (95% CI, 0.71-0.71), and PPV of 0.23 (95% CI, 0.21-0.25), correlating to an F1 score of 0.35 (95% CI, 0.35-0.35) and AUROC of 0.76 (95% CI, 0.75-0.77) (Table 2). Performance decreased compared with the training set, which had an AUROC of 0.93 (95% CI, 0.91-0.95) (Figure 2A). When analyzed by race, the performance displayed minimal variation: the AUROC range was 0.75 (95% CI, 95% CI, 0.72-0.78) for Asian patients and 0.75 (95% CI, 0.73-0.77) for Black patients to 0.77 (95% CI, 0.75-0.79) for patients with other or unknown race, and F1 score range was 0.32 (95% CI, 0.32-0.33) for White patients to 0.40 (95% CI, 0.39-0.42) for Black patients (Table 2 and Figure 2B).

**Table 2.** Performance Characteristics

Cohort	Positive rate	Total sample, No.	Sensitivity (95% CI)	Specificity (95% CI)	PPV, precision (95% CI)	NPV (95% CI)	Accuracy (95% CI)	F1 score (95% CI)	AUROC (95% CI)
Test set	0.11	43 274	0.71 (0.69-0.73)	0.71 (0.71-0.71)	0.23 (0.21-0.25)	0.95 (0.95-0.95)	0.71 (0.70-0.71	0.35 (0.35-0.35)	0.76 (0.75-0.77)
Asian	0.09	4112	0.70 (0.64-0.76)	0.70 (0.68-0.72)	0.19 (0.15-0.23)	0.96 (0.96-0.96)	0.70 (0.68-0.71)	0.30 (0.28-0.31)	0.75 (0.72-0.78)
Black	0.15	8180	0.72 (0.68-0.76)	0.68 (0.66-0.70)	0.28 (0.24-0.32)	0.93 (0.93-0.93)	0.69 (0.68-0.70)	0.40 (0.39-0.42)	0.75 (0.73-0.77)
White	0.10	22 580	0.70 (0.68-0.72)	0.72 (0.72-0.72)	0.21 (0.19-0.23)	0.95 (0.95-0.95)	0.71 (0.71-0.72)	0.32 (0.32-0.33)	0.76 (0.75-0.77)
Other or unknown^a^	0.11	8301	0.71 (0.67-0.75)	0.72 (0.70-0.74)	0.25 (0.21-0.29)	0.95 (0.95-0.95)	0.72 (0.71-0.73)	0.37 (0.36-0.38)	0.77 (0.75-0.79)

Abbreviations: AUROC, area under the receiver operating characteristic curve; NPV, negative predictive value; PPV, positive predictive value.

^a^ Other race was not broken down further in the database accessed. The Native American cohort was small (n = 45) and, thus, was excluded from further analysis.

Fairness metrics evaluated on all 6 pairwise comparisons for the 4 assessed racial subgroups satisfied having equal opportunity, equalized odds, and no disparate impact using our threshold range of 0.80 to 1.25 (1/0.8) (Table 3 and eFigure in Supplement 1). Estimated equal opportunity metrics between Asian patients compared with White individuals was 0.99 (95% CI, 0.97-1.01), Black patients compared with White patients was 0.96 (95% CI, 0.95-0.98), White patients compared with patients with other or unknown race was 0.98 (95% CI, 0.96-0.99), Asian patients compared with Black patients was 0.97 (95% CI, 0.95-0.99), and Black patients compared with patients with other or unknown race was 1.02 (95% CI, 1.00-1.04), all within the range of 0.80 to 1.25. Similarly, the estimated equalized odds and disparate impact values fell within the threshold range of 0.80 to 1.25. Since choice of threshold is arbitrary, we also considered an extreme threshold of 0.90 to 1.11 (1/0.9) and noted mild deviations from these ranges for only a few comparisons (equalized odds: Black patients compared with White patients, 0.87 [95% CI, 0.85-0.92]; Asian patients compared with patients with other or unknown race, 1.10 [95% CI, 1.03-1.16]; Black patients compared with patients with other or unknown race, 1.16 [95% CI, 1.10-1.21]; disparate impact: Black patients compared with White patients, 0.86 [95% CI, 0.82-0.89]; Black patients compared with patients with other or unknown race, 1.17 [95% CI, 1.12-1.22]).

**Table 3.** Fairness Metrics Comparisons Across Races

Racial category comparison	Ratio (95% CI)		
	Equal opportunity	Equalized odds	Disparate impact
Asian compared with Black	0.97 (0.95-0.99)	0.95 (0.90-1.00)	0.90 (0.85-0.94)
Asian compared with White	0.99 (0.97-1.01)	0.93 (0.89-0.98)	0.96 (0.91-1.00)
Asian compared with other^a^	0.99 (0.96-1.01)	1.10 (1.03-1.16)	1.04 (0.99-1.10)
Black compared with White	0.96 (0.95-0.98)	0.87 (0.85-0.92)	0.86 (0.82-0.89)
Black compared with other^a^	1.02 (1.00-1.04)	1.16 (1.10-1.21)	1.17 (1.12-1.22)
White compared with other^a^	0.98 (0.96-0.99)	1.03 (0.99-1.07)	1.00 (0.96-1.04)

^a^ Other race was not broken down further in the database accessed.

## Discussion

This study comprehensively assessed potential racial bias in a model predicting mortality for patients with solid tumors. In anticipation of clinical implementation, the AUROC and F1 score metrics demonstrated good agreement across the 4 analyzed racial subgroups, indicating similar performance for all groups (ie, no evidence of racial bias). Furthermore, all 6 comparisons of racial categories met all 3 fairness metrics within our safety threshold established prior to expected deployment. It is crucial to recognize that fairness metrics are descriptive tools for operational leaders, not prescriptive mandates. The decision to implement this model ultimately hinges on the interpretation of these metrics within the specific clinical context. While some numerical variation was observed in the fairness metric scores, all ratios remained within the prespecified range of 0.80 to 1.25 (1/0.8), suggesting that the prediction model is fair.

Our workflow prioritized achieving equal opportunity over the stricter equalized odds criterion if the latter proved to be impractical. A positive prediction triggers an SIC, which focuses on understanding the patient’s goals of care. False positives leading to SICs for low-risk patients are unlikely to cause harm, as the intervention is merely a conversation. Furthermore, the prediction is meant to supplement clinical judgment, not to replace it. Clinicians can initiate conversations with any patient for whom they deem the conversation to be necessary regardless of the model output. Therefore, a slight discrepancy in FPRs across race categories might not be significantly detrimental in this context.

However, a potential long-term risk with consistently higher FPRs for certain race categories could result in deleterious impacts. Since the workflow allows clinicians to act on the prediction and decide if they will initiate an SIC, it is possible that a consistently higher FPR in a subgroup may lead to alert fatigue, causing clinicians to ignore the output in the future. Conversely, a false-negative result could lead to denial of crucial end-of-life care, a more severe consequence. Since the equal opportunity score focuses on equal TPRs among groups, it also implies equal false-negative rates, making it potentially more relevant than equalized odds in this scenario. While our study showed minimal variations in equal opportunity ratios between racial groups, clinician alert fatigue from false-positive results remains an area for continued monitoring as the model is implemented.

When assessing clinical data, disparate impact may not be a practical target, as variances observed could potentially reflect actual structural issues in the health care system. While disparate impact ensures equal resource allocation, our study found minimal variation between subgroups. If a more stringent threshold was used, it should be noted that comparison with Black patients may not satisfy disparate impact. This warrants further investigation, as data patterns may differ among racial groups, potentially rendering the measure unreliable. Our mortality risk model suggests that certain groups may have higher baseline risk for mortality due to factors like access to care and structural racism. Measuring structural racism is complex, and several approaches have been used.^25,26,27^ These include a geographic approach examining segregation and poverty, a self-reported approach examining patient experiences of discrimination, a policy approach assessing contribution of health care policies leading to discrimination, and an outcomes approach examining outcomes such as prevalence and mortality. We are currently using an outcomes-based approach and have created a dashboard to monitor disparities in real time.

### Future

We will next implement the model into clinical workflows to trigger SICs. Healthcare utilization by identified patients will be tracked prospectively using a real-time dashboard identifying disparities at the aggregate level. In alignment with the recently proposed guiding principles to address the effect of algorithm bias on racial and ethnic disparities in health and health care,^28^ we plan to share this dashboard with our community stakeholders during implementation to ensure transparency and gather critical input.

### Strengths and Limitations

This study has strengths. It addressed a critical concern in health care AI: potential for algorithmic bias to perpetuate existing racial disparities. This study went beyond simply building a predictive model; it incorporated evaluations for racial bias throughout the development process. This included assessing fairness metrics, offering a deeper understanding of potential disparities beyond performance measures. Furthermore, analyzing subgroups and the relevance of the metrics to the intended clinical workflow ensured that the model performed fairly across different racial groups. By proactively evaluating for bias, we demonstrated a commitment to ethical deployment of the model in a clinical setting.

The study also has some limitations related to quality of data, especially pertaining to race. Death data from the Social Security Death Index experience a reporting time lag that prevents capture of recent deaths and has variable accuracy for patients from minoritized racial and ethnic groups.^29^ However, we obtained patients’ race from the cancer registry, which uses administrative databases, direct patient surveys, and imputed algorithms to obtain race data.^30^ Our dataset reported that 19.2% of patients were classified as other or unknown race. Although the racial bias assessments have utility, they would not be generalizable to smaller racial cohorts that may comprise this category. For model training, we excluded race as a predictor variable to minimize racial bias in the training phase. For many models, this is not sufficient to remove bias, as correlations may exist between race and other covariates that would continue to bias the model.^9^ Given that there were some statistical deviations to some fairness metrics, we may consider applying calibration methods in the future to further enhance the model.

## Conclusions

In this cohort study of patients included in ML model development, there was no significant bias among racial subgroups, as evidenced by minimal variation in model performance and selected fairness metrics. While there were some variations in fairness metrics, they were within the prespecified thresholds. These results should be interpreted in the context of the clinical implementation of the tool, which may warrant prioritizing one metric over another. The findings suggest that incorporating racial bias assessments into model development is feasible and should be the standard for reporting on tools intended for clinical practice.

## Notes

Supplement 1. **eTable.** Summary of Data Elements and Data Sources **eFigure.** Fairness Metrics Comparisons Across Races

Supplement 2.
Data Sharing Statement
