# Supplementary material for: Fairness in Predicting Cancer Mortality Across Racial Subgroups
Source: JAMA Netw Open. 2024 Jul 10;7(7):e2421290. doi: 10.1001/jamanetworkopen.2024.21290 (PMC11238025; doi:10.1001/jamanetworkopen.2024.21290)
Supplement: Supplement 1. — eTable. Summary of Data Elements and Data Sources eFigure. Fairness Metrics Comparisons Across Races [file jamanetwopen-e2421290-s001.pdf]

# Supplementary Online Content

Ganta T, Kia A, Parchure P, et al. Fairness in predicting cancer mortality across racial subgroups. *JAMA Netw Open*. 2024;7(7):e2421290. doi:10.1001/jamanetworkopen.2024.21290

**eTable.** Summary of Data Elements and Data Sources
**eFigure.** Fairness Metrics Comparisons Across Races

This supplementary material has been provided by the authors to give readers additional information about their work.

© 2024 Ganta T et al. *JAMA Network Open*.

## eTable: Summary of data elements and data sources

| Model Inputs | Data Source | Data Type | Number of Measurements Sampled |
|---|---|---|---|
| AGE | ADT Platform | Numerical | NA |
| ALBUMIN | Lab Platform | Numerical | 3 |
| AMYLASE | Lab Platform | Numerical | 1 |
| BILIRUBIN DIRECT | Lab Platform | Numerical | 1 |
| BLOOD CULTURE | Lab Platform | Categorical | 1 |
| BMI | Derived Variable | Numerical | 3 |
| BREATH SOUNDS BILATERAL | EHR (Nursing Flowsheet) | Categorical | 1 |
| BUN | Lab Platform | Numerical | 3 |
| CALCIUM | Lab Platform | Numerical | 3 |
| CHLORIDE | Lab Platform | Numerical | 3 |
| CREATININE | Lab Platform | Numerical | 3 |
| DIASTOLIC BLOOD PRESSURE | EHR (Vital Sign Flowsheet) | Numerical | 3 |
| EDEMA LEVEL | EHR (Nursing Flowsheet) | Categorical | 1 |
| GAMMA GTP BLD | Lab Platform | Numerical | 1 |
| HEMOGLOBIN | Lab Platform | Numerical | 3 |
| INR | Lab Platform | Numerical | 3 |
| NUMBER OF INPATIENT ADMISSIONS | Derived Variable | Numerical | NA |
| LIPASE | Lab Platform | Numerical | 1 |
| LYMPHOCYTE PERCENT | Lab Platform | Numerical | 1 |
| GENDER | ADT Platform | Categorical | NA |
| O2 SATURATION | EHR (Vital Sign Flowsheet) | Numerical | 3 |
| PAIN | EHR (Nursing Flowsheet) | Numerical | 3 |
| PHOSPHORUS BLOOD | Lab Platform | Numerical | 1 |
| PLATELET | Lab Platform | Numerical | 3 |
| POTASSIUM | Lab Platform | Numerical | 3 |
| PROTEIN TOTAL | Lab Platform | Numerical | 3 |
| PTT | Lab Platform | Numerical | 3 |
| PULSE | EHR (Vital Sign Flowsheet) | Numerical | 3 |

© 2024 Ganta T et al. *JAMA Network Open*.

| | | | |
|---|---|---|---|
| RESPIRATIONS | EHR (Vital Sign Flowsheet) | Numerical | 3 |
| SODIUM | Lab Platform | Numerical | 3 |
| SYSTOLIC BLOOD PRESSURE | EHR (Vital Sign Flowsheet) | Numerical | 3 |
| TEMPERATURE ORAL | EHR (Vital Sign Flowsheet) | Numerical | 3 |
| WBC | Lab Platform | Numerical | 3 |
| TREATMENT PLAN | EHR | Categorical | 1 |
| CANCER STAGE | EHR | Categorical | 1 |

© 2024 Ganta T et al. *JAMA Network Open*.

## eFigure: Fairness Metrics Comparisons Across Races

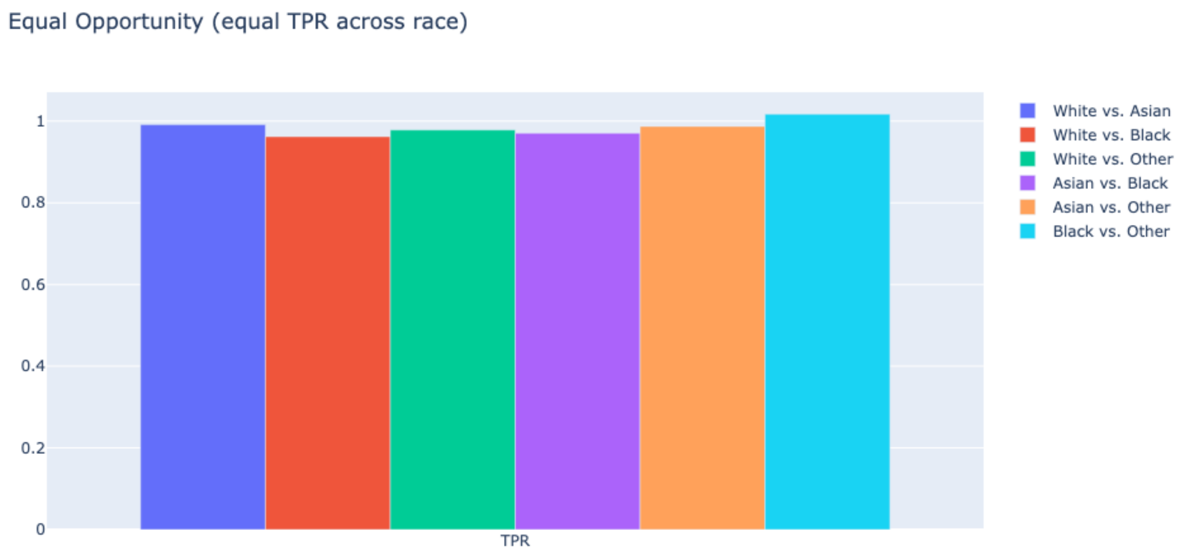


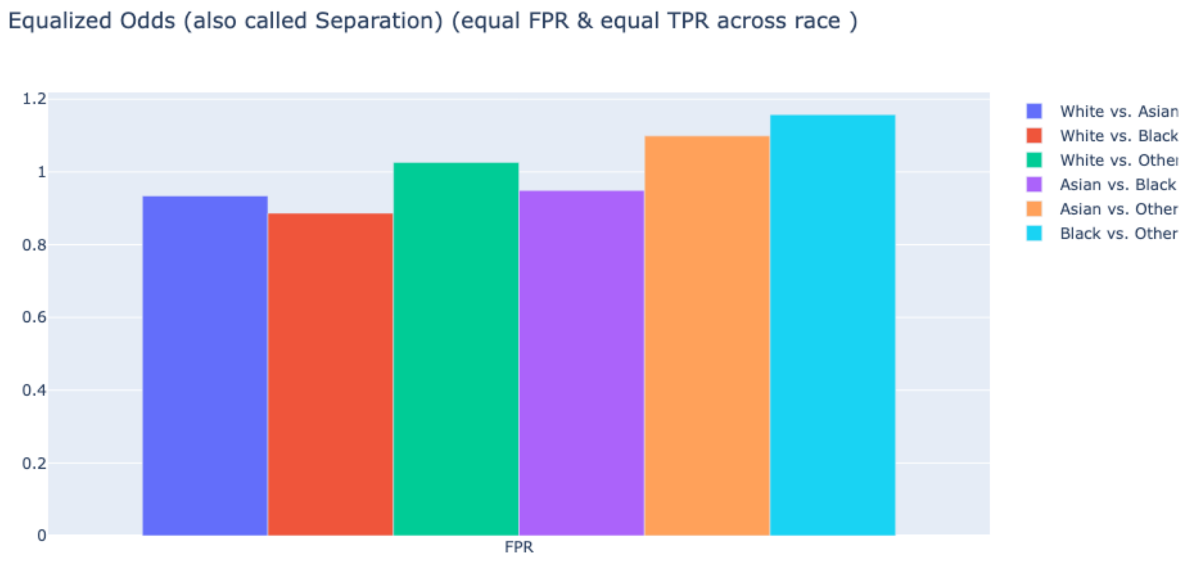


© 2024 Ganta T et al. *JAMA Network Open*.

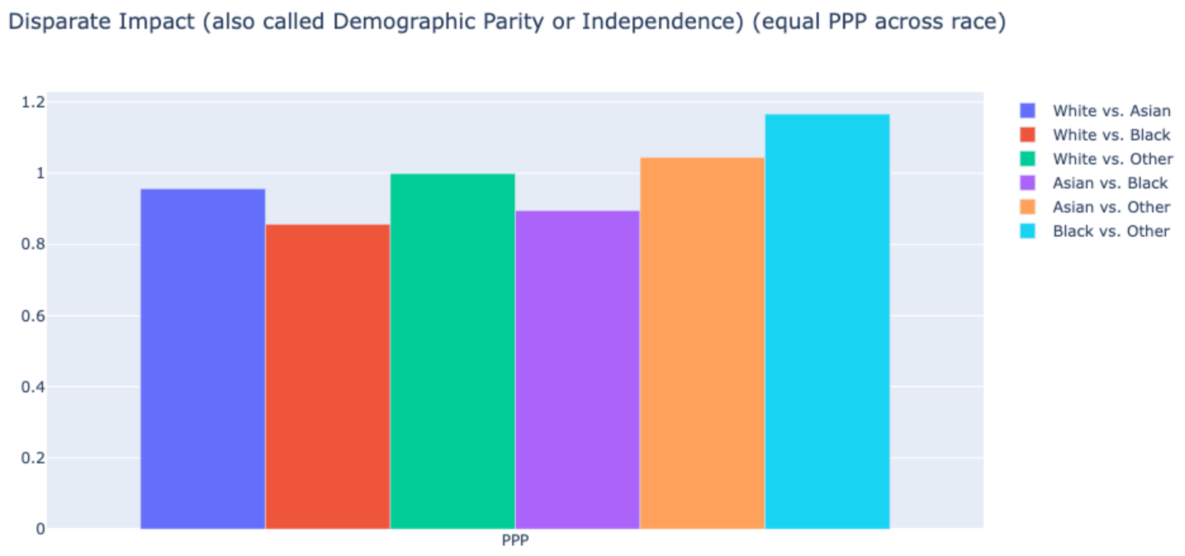


© 2024 Ganta T et al. *JAMA Network Open*.
