# Supplementary material for: Fairness in Predicting Cancer Mortality Across Racial Subgroups
Source: JAMA Netw Open. 2024 Jul 10;7(7):e2421290. doi: 10.1001/jamanetworkopen.2024.21290 (PMC11238025; doi:10.1001/jamanetworkopen.2024.21290)
Supplement: Supplement 2. — Data Sharing Statement [file jamanetwopen-e2421290-s002.pdf]

Data Sharing Statement

Ganta. Fairness in Predicting Cancer Mortality Across Racial Subgroups. *JAMA Netw Open*. Published July 10, 2024. doi:10.1001/jamanetworkopen.2024.21290

Data
Data available: No
